# Position Tracking of Multiple Robotic Manipulator Systems Associated with Communication Strength Dynamics

**DOI:** 10.3390/s23229275

**Published:** 2023-11-20

**Authors:** Juanxia Zhao, Yinhe Wang, Peitao Gao, Shengping Li, Haoguang Chen

**Affiliations:** 1 School of Automation, Guangdong University of Technology, Guangzhou 510006, China; 1112104030@mail2.gdut.edu.cn (J.Z.); yinhewang@gdut.edu.cn (Y.W.); 2 School of Electronics and Information, Guangdong Polytechnic Normal University, Guangzhou 510006, China; peitaogao@gpnu.edu.cn; 3 MOE Key Laboratory of Intelligent Manufacturing, Shantou University, Shantou 515063, China; 4 College of Computing, University of Electronic Science and Technology of China, Zhongshan Institute, Zhongshan 528402, China; haog_chen@zsc.edu.cn

**Keywords:** multiple robotic manipulator systems, robotic manipulator subsystem, communication strength subsystem, position tracking control

## Abstract

In general, a multiple robotic manipulator system (MRMS) with uncertainties can be considered a composition system with a robotic manipulator subsystem (RMS) and a communication strength subsystem (CSS), and both subsystems are coupled to each other. In this paper, a new position tracking control scheme is proposed for the MRMS while considering the communication strength dynamics between robotic manipulators. The control scheme designed in this paper consists of two parts: the first part is to design the control protocol in the RMS, and the second part is to design the coupling relationship in the CSS. Through these two parts, we can achieve the position tracking of an MRMS. Firstly, the dynamical mathematical model of the RMS and CSS in the MRMS is constructed, and the corresponding assumptions are given. Then, the corresponding stability analysis is proposed, which provides the basis for a theoretical understanding of the underlying problem. Finally, an illustrative example is presented to verify the effectiveness of the proposed control scheme.

## 1. Introduction

There has been increasing demand for multiple robotic manipulator systems (MRMSs) employed in modern manufacturing processes, such as assembling, transporting, painting, welding, and so on [1,2,3,4]. These applications require great manipulability and maneuverability; most of them cannot be achieved using a single robotic manipulator subsystem. In these situations, the utilization of multiple robotic manipulator systems is an effective choice [5,6,7,8,9,10].

From the perspective of a composition system, the MRMS with uncertainties can be considered a composition system with a robotic manipulator subsystem (RMS) and a communication strength subsystem (CSS), and both subsystems are coupled to each other. It is worth noting that the existing studies on MRMSs mainly focus on synchronization and consensus [11,12,13,14,15] for the RMS, while the communication strength between robotic manipulators plays only a secondary role. For example, in the work [12], Sun proposed adaptive controllers and a parameter estimator employing coupling control to achieve the position synchronization of multiple motion axes. In the work [16], a distributed synchronization control scheme was proposed for a group of MRMSs with model uncertainties subject to time-varying communication delays. In the work [17], a T-S(Takagi–Sugeno) adaptive tracking algorithm control based on the small gain theorem was proposed for an uncertain MRMS. In the work [18], a novel adaptive control scheme for an MRMS with a time-varying parameter was proposed based on the radial basis function neural network. From the perspective of the composition system, the dynamical behavior of an RMS can be affected by the dynamical behavior of a CSS in the MRMS, and vice versa. However, in the works [11,12,13,14,15,16,17,18], the communication strength between robotic manipulators was not considered as a subsystem to be studied. In other words, it has been rare to study the dynamics of a CSS that is considered a subsystem with dynamic behavior. In summary, how to design a tracking control protocol for the RMS and a coupling matrix function for the CSS while considering the uncertainties in MRMSs is an interesting and challenging issue for the position tracking of MRMSs.

Inspired by the literature [19,20,21,22], the dynamical model of an RMS is shown mathematically as the vector differential equation with the second derivative term due to using Newton’s law of motion. Furthermore, the dynamical model of a CSS is shown mathematically as the vector differential equation with outgoing communication strength characteristics. In recent years, there have been a number of studies [20,21,22,23] on complex dynamic networks in which vector differential equations have been used to model the dynamics of the outgoing links. For example, in [21], the concept of outgoing links was introduced using the idea of ego networks, and the dynamics of the outgoing links were modeled using vector differential equations. In the RMS considered in this paper, each robotic manipulator can be regarded as a node, and the information communication between robotic manipulators can be regarded as the outgoing link. Inspired by this, vector differential equations are considered in modeling the dynamics of the CSS. To sum up, the motivation of this study is to develop a new tracking control protocol for an RMS and coupling matrix function for a CSS such that the position of an MRMS can be tracked on the desired joint position trajectory.

Compared to the literature [12,13,14,15,16,17,18,21,22], the main contributions and innovations of this paper include the following three points.

(i) The dynamical model of an RMS is described using a vector differential equation with the second derivative term and interconnected term due to Newton’s law of motion; the model is more general.
(ii) A vector differential equation is used to model the dynamics of CSS, and there are few studies considering the variation of the strength of communication between multiple robotic manipulators in the existing literature.
(iii) The position tracking for an MRMS is achieved by employing the position tracking control protocol for the RMS and the coupling matrix function for the CSS designed in this paper, which has been rarely reported in existing studies.

This paper is organized as follows. In Section 2, the dynamical models of an MRMS with an RMS and CSS are presented, and the corresponding assumptions are given. In Section 3, the control goal of this paper is put forward, and the corresponding control protocol and the coupling matrix function are synthesized. In Section 4, the effectiveness of the result obtained in this paper is verified via MATLAB numerical simulation for the *N* continuous two-links robotic manipulator. Finally, the conclusions of this work and the future directions are discussed in Section 5.

Notations: $diag(b_{1},b_{2},\cdots ,b_{n})$ denotes a diagonal matrix with $b_{1},b_{2},\cdots ,b_{n}$ as diagonal elements; $∥\cdot ∥$ denotes the Euclidean norm of vector “·” or the Frobenius norm of matrix “·”; $R^{n}$ denotes an *n*-dimensional Euclidean space; $R^{n\times m}$ denotes n×m real matrices; randn(1) denotes the generation of a random number with a standard normal distribution; rand(a,b) denotes the generation of a matrix of size a×b, where each element is a random number in the interval [0, 1].

## 2. Model Description and Control Design

Consider the uncertain multiple robotic manipulator systems (MRMSs) consisting of *N* robotic manipulators with the second derivative term, $p_{ij}\left(t\right)\in R$, denoting the communication strength of the *i*th robotic manipulator pointing to the *j*th robotic manipulator. If $p_{ij}\left(t\right)=p_{ji}\left(t\right)$, then the multiple robotic manipulators system is undirected, in which $p_{ii}\left(t\right)$ denotes the communication strength of the self-link and $i,j\in \left\{1,2,\ldots ,N\right\}$. The dynamical equation of the *i*th robotic manipulator subsystem (RMS) is represented as follows.

(1) $$D_{i}\left(z_{i}\right){\ddot{z}}_{i}+C_{i}\left(z_{i},{\dot{z}}_{i}\right){\dot{z}}_{i}+G_{i}\left(z_{i}\right)+\varepsilon_{i}\left(z_{i},{\dot{z}}_{i}\right)=\tau_{i}-\sigma \sum_{j=1}^{N}p_{ij}\left(t\right)\Gamma h_{j}\left(z\right),$$

where $z_{i}={\left(z_{i1},z_{i2},\ldots ,z_{in}\right)}^{T}\in R{}^{n}$ and ${\dot{z}}_{i}={\left({\dot{z}}_{i1},{\dot{z}}_{i2},\ldots ,{\dot{z}}_{in}\right)}^{T}\in R{}^{n}$ denote the joint angular position vector and the velocity vector of the *i*th robotic manipulator, respectively; ${\ddot{z}}_{i}={\left({\ddot{z}}_{i1},{\ddot{z}}_{i2},\ldots ,{\ddot{z}}_{in}\right)}^{T}\in R{}^{n}$ denotes the acceleration vector of the *i*th robotic manipulator; $z={\left(z_{1}^{T},z_{2}^{T},\ldots ,z_{N}^{T}\right)}^{T}\in R{}^{nN}$ denotes the overall position vector of the RMS; σ>0 is the common coupling strength of the *i*th RMS; $D_{i}\left(z_{i}\right)\in R{}^{n\times n}$, $C_{i}\left(z_{i},{\dot{z}}_{i}\right)\in R{}^{n\times n}$, and $G_{i}\left(z_{i}\right)\in R{}^{n}$ denote the inertia matrix, the Coriolis and centrifugal torque matrix, and the gravitational force vector, respectively; $\varepsilon_{i}\left(z_{i},{\dot{z}}_{i}\right)\in R{}^{n}$ denotes the appropriate dimensioned uncertain vector of the *i*th robotic manipulator; and $\tau_{i}\in R{}^{n}$ and $\sum_{j=1}^{N}p_{ij}\left(t\right)\Gamma h_{j}\left(z\right)$ denote the designable control protocol of *i*th robotic manipulator and the determined communication protocol between the RMS and the CSS, respectively, in which $\Gamma =diag\left(b_{1},b_{2},...,b_{n}\right)\in R{}^{n\times n}$ is the diagonal matrix with the constants $b_{1},b_{2},...,b_{n}$ and $h_{j}\left(z\right)\in R{}^{n}$ is the inner coupling vector function.

**Remark** **1.** *From the perspective of the composition system [24,25,26], the determined communication protocol $\sum_{j=1}^{N}p_{ij}\left(t\right)\Gamma h_{j}\left(z\right)$ in Equation (1) can be described as the coupled communication strength between the ith robotic manipulator and its adjacent robotic manipulator, in which $p_{ij}\left(t\right)$ is used to describe the information communication strength of the ith robotic manipulator pointing to the jth robotic manipulator for $i,j\in \left\{1,2,\ldots ,N\right\}$.*

From the perspective of engineering applications, Equation (1) describes the dynamics of the *i*th RMS, in which $p_{ij}\left(t\right)$ denotes the communication strength of the *i*th robotic manipulator pointing to the *j*th robotic manipulator. In other words, the dynamical change of $p_{ij}\left(t\right)$ influences the position information of the *i*th RMS. Inspired by the literature [21,22], $p_{ij}\left(t\right)$ is dynamically changing according to the following dynamical equation.

(2) $$\frac{dp_{ij}\left(t\right)}{dt}=\sum_{k=1}^{N}a_{jk}^{i}p_{ik}\left(t\right)+\sum_{\rho =1}^{n}\theta_{j\rho }^{i}\left(z_{i}\right)\left(z_{i\rho }\left(t\right)+{\dot{z}}_{i\rho }\left(t\right)\right),$$

where $A_{i}={\left(a_{jk}^{i}\right)}_{N\times N}\in R{}^{N\times N}$, $\Theta_{i}\left(z_{i}\right)={\left(\theta_{j\rho }^{i}\left(z_{i}\right)\right)}_{N\times n}\in R{}^{N\times n}$, $i,j,k\in \left\{1,2,\ldots ,N\right\}$, and $\rho \in \left\{1,2,\ldots ,n\right\}$.

**Remark** **2.** *Equation (2) consists mainly of the linear part $a_{jk}^{i}p_{ik}\left(t\right)$ and the compensation part $\theta_{j\rho }^{i}\left(z_{i}\right)\left(z_{i\rho }\left(t\right)+{\dot{z}}_{i\rho }\left(t\right)\right)$, in which $i,j,k\in \left\{1,2,\ldots ,N\right\}$ and $\rho \in \left\{1,2,\ldots ,n\right\}$. Equation (2) can be explained graphically as follows.*

**Figure 1:**
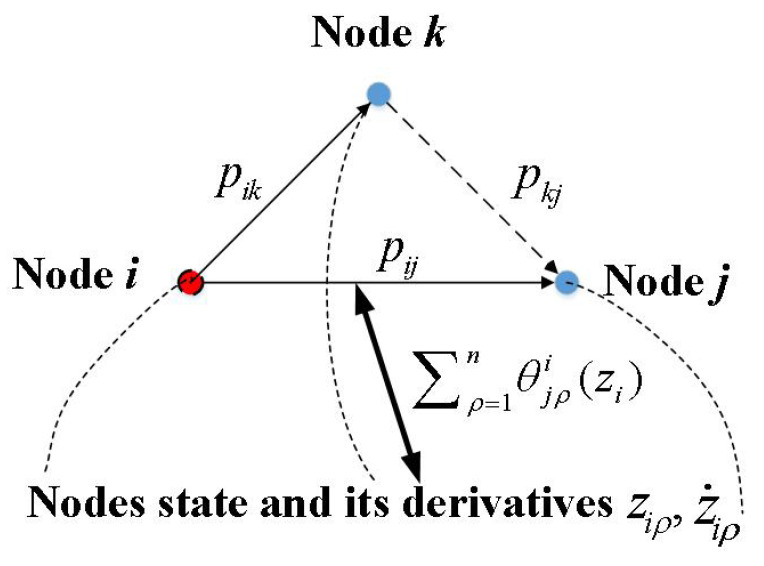
The schematic diagram of $p_{ij}\left(t\right)$ influenced by $p_{ik}\left(t\right)$, $z_{i\rho }\left(t\right)$ and ${\dot{z}}_{i\rho }\left(t\right)$ in Equation (2).

In Figure 1, for a concise representation, each node denotes a robotic manipulator in the MRMS. It can be observed from Figure 1 that the communication strength $p_{ij}\left(t\right)$ (which denotes the communication strength of the *i*th robotic manipulator pointing to the *j*th robotic manipulator) is regarded as being affected directly by $p_{ik}\left(t\right)$ (with the help of the $p_{kj}\left(t\right)$), $\theta_{j\rho }^{i}\left(z_{i}\right)$, $a_{jk}^{i}$, $z_{i\rho }$, and ${\dot{z}}_{i\rho }$. In other words, the communication strength $p_{ij}\left(t\right)$ is regarded as the linear operation of $p_{ik}\left(t\right)$, $z_{i\rho }$, and ${\dot{z}}_{i\rho }$ for all $i,j,k\in \left\{1,2,\ldots ,N\right\}$ and $\rho \in \left\{1,2,\ldots ,n\right\}$.

**Definition** **1** ([21,22]). *$P_{i}\left(t\right)={\left(\begin{array}{cccc} p_{i1}\left(t\right) & p_{i2}\left(t\right) & \ldots & p_{iN}\left(t\right) \end{array}\right)}^{T}\in R{}^{N}$ is called the outgoing communication strength vector for the ith robotic manipulator, respectively, $i\in \left\{1,2,\ldots ,N\right\}$.*

**Remark** **3.** *$p_{ij}$ represents the intensity of information transmission between the ith robotic manipulator and the jth robotic manipulator, and $P_{i}$, which is composed of all $p_{ij}$, $j\in \left\{1,2,\ldots ,N\right\}$, represents the strength of the information communication between the ith robotic manipulator and all other robotic manipulators. Then, all $P_{i}$, $i\in \left\{1,2,\ldots ,Nm\right\}$ consist of the communication strength subsystem (CSS).*

Note that $H_{i}\left(z\right)=\left(\begin{array}{cccc} h_{1}\left(z\right) & h_{2}\left(z\right) & \ldots & h_{N}\left(z\right) \end{array}\right)\in R{}^{n\times N}$, and then, Equation (1) can be formulated as

(3) $$D_{i}\left(z_{i}\right){\ddot{z}}_{i}+C_{i}\left(z_{i},{\dot{z}}_{i}\right){\dot{z}}_{i}+G_{i}\left(z_{i}\right)+\varepsilon_{i}\left(z_{i},{\dot{z}}_{i}\right)=\tau_{i}-\sigma \Gamma H_{i}\left(z\right)P_{i}\left(t\right).$$

**Assumption** **1.** *For Equation (3), $D_{i}\left(z_{i}\right)$, $C_{i}\left(z_{i},{\dot{z}}_{i}\right)$ are known matrices, and $G_{i}\left(z_{i}\right)$ is a known vector, in which $D_{i}\left(z_{i}\right)$ is a symmetric, bounded invertible matrix for all $z_{i}\in R{}^{n}$. Moreover, $H_{i}\left(z\right)$ is a known and bounded matrix, and the uncertain vector $\varepsilon_{i}\left(z_{i},{\dot{z}}_{i}\right)$ satisfies $∥\varepsilon_{i}\left(z_{i},{\dot{z}}_{i}\right)∥\leq \omega \left(t\right)$, where ω(t) represents the known positive function and $∥*∥$ represents the Euclidean norm of vector or matrix.*

**Assumption** **2.** *For Equation (3), the position $z_{i}\in R{}^{n}$ and the velocity ${\dot{z}}_{i}\in R{}^{n}$ of the ith robotic manipulator are available.*

**Remark** **4.** *In Equation (3), the outgoing communication strength vector $P_{i}\left(t\right)\in R{}^{N}$ denotes the overall communication strength set of the ith robotic manipulator pointing to the other robotic manipulator. In addition, Assumption 2 is mainly enlightened by some practical engineering systems. For example, in multiple robotic manipulator systems [12,13,14,15,16,27,28,29], $z_{i}\in R{}^{n}$ and ${\dot{z}}_{i}\in R{}^{n}$ represent the position vector of the ith robotic manipulator and the velocity vector of the ith robotic manipulator, respectively. Their state variables, such as the angle of each joint and the speed of each joint movement, can be measured by sensors.*

From the perspective of the outgoing communication strength vector, Equation (2) can be rewritten as follows.

(4) $${\dot{P}}_{i}\left(t\right)=A_{i}P_{i}\left(t\right)+\Theta_{i}\left(z_{i}\right)\left(z_{i}\left(t\right)+{\dot{z}}_{i}\left(t\right)\right),$$

in which $A_{i}\in R{}^{N\times N}$ denotes the appropriate dimensioned constant matrix and $\Theta_{i}\left(z_{i}\right)\in R{}^{N\times n}$ denotes the coupling matrix function between the CSS and RMS, where $i\in \left\{1,2,\ldots ,N\right\}$.

**Assumption** **3.** *For Equation (4), the $A_{i}$ is a Hurwitz matrix for all $i\in \left\{1,2,\ldots ,N\right\}$.*

It is noted from Assumption 3 that all eigenvalues of $A_{i}$ are located in the left half-plane. From the Lyapunov theory, it can be observed that there exist positive definite matrices $M_{i}\in R{}^{N\times N}$ and $Q_{i}\in R{}^{N\times N}$, which satisfy the following Lyapunov Equation (5), $i\in \left\{1,2,\ldots ,N\right\}$.

(5) $$A_{i}^{T}M_{i}+M_{i}A_{i}=-Q_{i}.$$

**Remark** **5.** *(i) For Equation (4), $\Theta_{i}\left(z_{i}\right)\in R{}^{N\times n}$ denotes the coupling matrix function between the RMS and CSS and needs to be designed. (ii) Equation (4) is largely enlightened by the literature [21,22], which represents the dynamical properties of the links relationship (communication strength) between nodes (robotic manipulators) in the complex dynamical network, where $P_{i}\left(t\right)\in R{}^{N}$ denotes the outgoing link vector (outgoing communication strength vector) of the ith node (robotic manipulator) for all $i\in \left\{1,2,\ldots ,N\right\}$.*

## 3. The Design of Tracking Control Protocol and Coupling Matrix Function

We introduce $z_{i}^{d}=z_{i}^{d}\left(t\right)\in R{}^{n}$ and $P_{i}^{d}=P_{i}^{d}\left(t\right)\in R{}^{N}$, which are the desired joint position trajectory of the *i*th robotic manipulator and the desired communication strength trajectory of the *i*th CSS, respectively. In addition, $z_{i}^{d}\left(t\right)\in R{}^{n}$ and its derivatives ${\dot{z}}_{i}^{d}\left(t\right)\in R{}^{n}$, ${\ddot{z}}_{i}^{d}\left(t\right)\in R{}^{n}$ are all bounded and smooth. Note that $e_{i}\left(t\right)=z_{i}\left(t\right)-z_{i}^{d}\left(t\right)$ and $E_{i}\left(t\right)=P_{i}\left(t\right)-P_{i}^{d}\left(t\right)$ denote the position tracking error of the *i*th robotic manipulator and the communication strength error of the *i*th CSS, respectively, where $i\in \left\{1,2,\ldots ,N\right\}$.

Control goal. Consider the MRMS with RMS (3) and CSS (4). Design the control protocol $\tau_{i}$ for RMS (3) and the coupling matrix function $\Theta_{i}\left(z_{i}\right)\in R{}^{N\times n}$ for CSS (4) such that $e_{i}\left(t\right)=z_{i}\left(t\right)-z_{i}^{d}\left(t\right)$$\overset{t\to +\infty }{\to }$ 0 holds, which means that the multiple robotic manipulator system achieves position tracking. Meanwhile, the outgoing communication strength vector $P_{i}=P_{i}\left(t\right)\in R{}^{N}$ is bounded. In order to achieve the above control goal, the following control scheme is obtained.

(6) $$\tau_{i}=D_{i}\left(z_{i}\right)\left\{{\ddot{z}}_{i}^{d}\left(t\right)-{\dot{e}}_{i}\left(t\right)-e_{i}\left(t\right)+\eta_{i}\right\}+C_{i}\left(z_{i},{\dot{z}}_{i}\right){\dot{z}}_{i}\left(t\right)+G_{i}\left(z_{i}\right)+\sigma \Gamma H_{i}\left(z\right)P_{i}^{d}\left(t\right),$$

(7) $$\eta_{i}=-∥D_{i}^{-1}\left(z_{i}\right)∥\omega \left(t\right)\tilde{sign}\left({\dot{e}}_{i}\left(t\right)+e_{i}\left(t\right)\right),$$

(8) $$\Theta_{i}\left(z_{i}\right)=\beta \sigma M_{i}^{-1}D_{i}^{-1}\left(z_{i}\right)\Gamma H_{i}\left(z\right),$$

where $I_{n}\in R{}^{n\times n}$ denotes the *n*th order identity matrix and β>0 denotes an adjustable positive parameter, where $i\in \left\{1,2,\ldots ,N\right\}$. $\tilde{sign}\left({\dot{e}}_{i}\left(t\right)+e_{i}\left(t\right)\right)=\left\{\begin{array}{c} \frac{{\dot{e}}_{i}\left(t\right)+e_{i}\left(t\right)}{∥{\dot{e}}_{i}^{T}\left(t\right)+e_{i}^{T}\left(t\right)∥},{\dot{e}}_{i}\left(t\right)+e_{i}\left(t\right)\neq 0\\ 0,{\dot{e}}_{i}\left(t\right)+e_{i}\left(t\right)=0 \end{array}\right.$, and it is seen that $\left({\dot{e}}_{i}^{T}\left(t\right)+e_{i}^{T}\left(t\right)\right)\cdot \tilde{sign}\left({\dot{e}}_{i}\left(t\right)+e_{i}\left(t\right)\right)=∥{\dot{e}}_{i}^{T}\left(t\right)+e_{i}^{T}\left(t\right)∥$ holds.

In addition, in order to assist the RMS in achieving positional tracking, an auxiliary communication strength $P_{i}^{d}\left(t\right)\in R{}^{N}$ is introduced, and the dynamical equation of $P_{i}^{d}\left(t\right)\in R{}^{N}$ can be described as follows

(9) $${\dot{P}}_{i}^{d}\left(t\right)=A_{i}P_{i}^{d}\left(t\right)+\Theta_{i}\left(z_{i}\right)\left(z_{i}^{d}\left(t\right)+{\dot{z}}_{i}^{d}\left(t\right)\right).$$

**Remark** **6.** *(i) For Equation (8), since $M_{i}$ is the positive definite matrix and $D_{i}\left(z_{i}\right)$ is a symmetric, bounded invertible matrix, that is, the inverse of matrices $M_{i}$ and $D_{i}\left(z_{i}\right)$ is bounded, β, σ, *Γ*, and $H_{i}\left(z\right)$ are bounded. It can be concluded that the coupling matrix function $\Theta_{i}\left(z_{i}\right)$ is bounded, where $i\in \left\{1,2,\ldots ,N\right\}$. (ii) In Equation (9), because $z_{i}^{d}\left(t\right)$, ${\dot{z}}_{i}^{d}\left(t\right)$, and $\Theta_{i}\left(z_{i}\right)$ are bounded, that is, $\Theta_{i}\left(z_{i}\right)\left(z_{i}^{d}\left(t\right)+{\dot{z}}_{i}^{d}\left(t\right)\right)$ is bounded, and $A_{i}$ is a Hurwitz matrix, it follows that the desired communication strength trajectory $P_{i}^{d}\left(t\right)$ is bounded, where $i\in \left\{1,2,\ldots ,N\right\}$. (iii) The control protocols (6) and (7) and the coupling matrix function (8) constitute the control scheme of this paper, where the control protocols (6) and (7) are designed in the RMS and the coupling relation (8) is designed in the CSS. The combined action of these two parts makes the position of the ith robotic manipulator track along the desired joint position trajectory, while the communication strength between robotic manipulators is bounded, where $i\in \left\{1,2,\ldots ,N\right\}$. (iv) In designing the control scheme, the information that can be used is the state information of the multiple robotic manipulators, such as the position, speed, acceleration of the multiple robotic manipulators, and some information that is known in the system. It is worth noting that we cannot utilize the communication strength information because the position and speed of the robotic manipulators can be measured via suitable sensors, whereas the strength of the communication between the robotic manipulators is difficult to be accurately measured using suitable sensors. Therefore, because of cost, technical limitations, and other factors, the strength of communication between robotic manipulators cannot be accurately obtained in this paper, and consequently, the information on communication strength $p_{i}$ cannot be used in the control scheme.*

From $E_{i}\left(t\right)=P_{i}\left(t\right)-P_{i}^{d}\left(t\right)$, Equations (3), (4) and (9), and the control protocol (6), the position tracking error and the communication strength error equations for the RMS (3) and CSS (4) can be obtained as follows:

(10) $${\ddot{e}}_{i}\left(t\right)+{\dot{e}}_{i}\left(t\right)+e_{i}\left(t\right)+\sigma D_{i}^{-1}\left(z_{i}\right)\Gamma H_{i}\left(z\right)E_{i}\left(t\right)+D_{i}^{-1}\left(z_{i}\right)\varepsilon_{i}\left(z_{i},{\dot{z}}_{i}\right)=\eta_{i}.$$

(11) $$\begin{array}{cc} {\dot{E}}_{i}\left(t\right) & =A_{i}P_{i}\left(t\right)+\Theta_{i}\left(z_{i}\right)\left[z_{i}\left(t\right)+{\dot{z}}_{i}\left(t\right)\right]-{\dot{P}}_{i}^{d}\left(t\right)\\ \; & =A_{i}\left(P_{i}\left(t\right)-P_{i}^{d}\left(t\right)\right)+\Theta_{i}\left(z_{i}\right)\left[z_{i}\left(t\right)-z_{i}^{d}\left(t\right)+{\dot{z}}_{i}\left(t\right)-{\dot{z}}_{i}^{d}\left(t\right)\right]\\ \; & =A_{i}E_{i}\left(t\right)+\Theta_{i}\left(z_{i}\right)\left[{\dot{e}}_{i}\left(t\right)+e_{i}\left(t\right)\right]. \end{array}$$

**Theorem** **1.** *Consider the MRMS with RMS (3) and CSS (4). If Assumptions 1–3 are satisfied, the control protocols $\tau_{i}$ and $\eta_{i}$ are designed, and the coupling matrix function $\Theta_{i}\left(z_{i}\right)$ is synthesized; the position tracking error $e_{i}\left(t\right)$ is asymptotically stable and the outgoing communication strength vector $P_{i}=P_{i}\left(t\right)\in R{}^{N}$ is bounded for all $i\in \left\{1,2,\ldots ,N\right\}$.*

**Proof** **of Theorem.** Consider the positive definite function $V_{i}\left(t\right)=V_{i}\left[e_{i}\left(t\right),\;{\dot{e}}_{i}\left(t\right),\;E_{i}\left(t\right)\right]=\beta \sum_{i=1}^{N}\left(e_{i}^{T}\left(t\right),\;{\dot{e}}_{i}^{T}\left(t\right)\right)\left[\begin{array}{cc} 2I_{n} & I_{n}\\ I_{n} & I_{n} \end{array}\right]\left(\begin{array}{c} e_{i}\left(t\right)\\ {\dot{e}}_{i}\left(t\right) \end{array}\right)+\sum_{i=1}^{N}E_{i}^{T}\left(t\right)M_{i}E_{i}\left(t\right)$, where since $\left[\begin{array}{cc} 2I_{n} & I_{n}\\ I_{n} & I_{n} \end{array}\right]$ and $M_{i}$ are positive definite matrices, it is clear that $V_{i}\left(t\right)$ is positive definite. To facilitate the subsequent derivation, it can be written in the vector form as follows:

(12) $$\begin{array}{cc} V_{i}\left(t\right) & =\beta \sum_{i=1}^{N}\left(e_{i}^{T}\left(t\right),{\dot{e}}_{i}^{T}\left(t\right)\right)\left[\begin{array}{cc} 2I_{n} & I_{n}\\ I_{n} & I_{n} \end{array}\right]\left(\begin{array}{c} e_{i}\left(t\right)\\ {\dot{e}}_{i}\left(t\right) \end{array}\right)+\sum_{i=1}^{N}E_{i}^{T}\left(t\right)M_{i}E_{i}\left(t\right)\\ \; & =2\beta \sum_{i=1}^{N}e_{i}^{T}\left(t\right)e_{i}\left(t\right)+\beta \sum_{i=1}^{N}{\dot{e}}_{i}^{T}\left(t\right){\dot{e}}_{i}\left(t\right)\\ \; & \;+2\beta \sum_{i=1}^{N}e_{i}^{T}\left(t\right){\dot{e}}_{i}\left(t\right)+\sum_{i=1}^{N}E_{i}^{T}\left(t\right)M_{i}E_{i}\left(t\right). \end{array}$$

Differentiating $V_{i}\left(t\right)$ with respect to time yields

(13) $$\begin{array}{cc} {\dot{V}}_{i}\left(t\right) & =4\beta \sum_{i=1}^{N}e_{i}^{T}\left(t\right){\dot{e}}_{i}\left(t\right)+2\beta \sum_{i=1}^{N}{\dot{e}}_{i}^{T}\left(t\right){\ddot{e}}_{i}\left(t\right)+2\beta \sum_{i=1}^{N}{\dot{e}}_{i}^{T}\left(t\right){\dot{e}}_{i}\left(t\right)\\ \; & \;+2\beta \sum_{i=1}^{N}e_{i}^{T}\left(t\right){\ddot{e}}_{i}\left(t\right)+2\sum_{i=1}^{N}E_{i}^{T}\left(t\right)M_{i}{\dot{E}}_{i}\left(t\right)\\ \; & =4\beta \sum_{i=1}^{N}e_{i}^{T}\left(t\right){\dot{e}}_{i}\left(t\right)+2\beta \sum_{i=1}^{N}\left({\dot{e}}_{i}^{T}\left(t\right)+e_{i}^{T}\left(t\right)\right){\ddot{e}}_{i}\left(t\right)\\ \; & \;+2\beta \sum_{i=1}^{N}{\dot{e}}_{i}^{T}\left(t\right){\dot{e}}_{i}\left(t\right)+2\sum_{i=1}^{N}E_{i}^{T}\left(t\right)M_{i}{\dot{E}}_{i}\left(t\right) \end{array}$$

According to Equations (10) and (11), Equation (13) can be derived as

$$\begin{array}{cc} {\dot{V}}_{i}\left(t\right) & =2\beta \sum_{i=1}^{N}\left({\dot{e}}_{i}^{T}\left(t\right)+e_{i}^{T}\left(t\right)\right)\left[\eta_{i}-{\dot{e}}_{i}\left(t\right)-e_{i}\left(t\right)-\sigma D_{i}^{-1}\left(z_{i}\right)\Gamma H_{i}\left(z\right)E_{i}\left(t\right)-D_{i}^{-1}\left(z_{i}\right)\varepsilon_{i}\left(z_{i},{\dot{z}}_{i}\right)\right]\\ \; & \;+4\beta \sum_{i=1}^{N}e_{i}^{T}\left(t\right){\dot{e}}_{i}\left(t\right)+2\beta \sum_{i=1}^{N}{\dot{e}}_{i}^{T}\left(t\right){\dot{e}}_{i}\left(t\right)\\ \; & \;+2\sum_{i=1}^{N}E_{i}^{T}\left(t\right)M_{i}\left\{A_{i}E_{i}\left(t\right)+\Theta_{i}\left(z_{i}\right)\left[{\dot{e}}_{i}\left(t\right)+e_{i}\left(t\right)\right]\right\}\\ \; & =4\beta \sum_{i=1}^{N}e_{i}^{T}\left(t\right){\dot{e}}_{i}\left(t\right)-2\beta \sum_{i=1}^{N}\left({\dot{e}}_{i}^{T}\left(t\right)+e_{i}^{T}\left(t\right)\right)\left({\dot{e}}_{i}\left(t\right)+e_{i}\left(t\right)\right)+2\beta \sum_{i=1}^{N}{\dot{e}}_{i}^{T}\left(t\right){\dot{e}}_{i}\left(t\right)\\ \; & \;+2\beta \sum_{i=1}^{N}\left({\dot{e}}_{i}^{T}\left(t\right)+e_{i}^{T}\left(t\right)\right)\left[\eta_{i}-\sigma D_{i}^{-1}\left(z_{i}\right)\Gamma H_{i}\left(z\right)E_{i}\left(t\right)-D_{i}^{-1}\left(z_{i}\right)\varepsilon_{i}\left(z_{i},{\dot{z}}_{i}\right)\right]\\ \; & \;+2\sum_{i=1}^{N}E_{i}^{T}\left(t\right)M_{i}A_{i}E_{i}\left(t\right)+2\sum_{i=1}^{N}E_{i}^{T}\left(t\right)M_{i}\Theta_{i}\left(z_{i}\right)\left[{\dot{e}}_{i}\left(t\right)+e_{i}\left(t\right)\right] \end{array}$$

According to Equation (5), it can be further obtained that

(14) $$\begin{array}{cc} {\dot{V}}_{i}\left(t\right) & =2\beta \sum_{i=1}^{N}\left({\dot{e}}_{i}^{T}\left(t\right)+e_{i}^{T}\left(t\right)\right)\left[\eta_{i}-\sigma D_{i}^{-1}\left(z_{i}\right)\Gamma H_{i}\left(z\right)E_{i}\left(t\right)-D_{i}^{-1}\left(z_{i}\right)\varepsilon_{i}\left(z_{i},{\dot{z}}_{i}\right)\right]\\ \; & \;-2\beta \sum_{i=1}^{N}e_{i}^{T}\left(t\right)e_{i}\left(t\right)-\sum_{i=1}^{N}E_{i}^{T}\left(t\right)Q_{i}E_{i}\left(t\right)+2\sum_{i=1}^{N}E_{i}^{T}\left(t\right)M_{i}\Theta_{i}\left(z_{i}\right)\left[{\dot{e}}_{i}\left(t\right)+e_{i}\left(t\right)\right]\\ \; & =-2\beta \sum_{i=1}^{N}e_{i}^{T}\left(t\right)e_{i}\left(t\right)-\sum_{i=1}^{N}E_{i}^{T}\left(t\right)Q_{i}E_{i}\left(t\right)\\ \; & \;+2\beta \sum_{i=1}^{N}\left({\dot{e}}_{i}^{T}\left(t\right)+e_{i}^{T}\left(t\right)\right)\left[\eta_{i}-D_{i}^{-1}\left(z_{i}\right)\varepsilon_{i}\left(z_{i},{\dot{z}}_{i}\right)\right]\\ \; & \;-2\beta \sigma \sum_{i=1}^{N}E_{i}^{T}\left(t\right)D_{i}^{-1}\left(z_{i}\right)\Gamma H_{i}\left(z\right)\left[{\dot{e}}_{i}\left(t\right)+e_{i}\left(t\right)\right]\\ \; & \;+2\sum_{i=1}^{N}E_{i}^{T}\left(t\right)M_{i}\Theta_{i}\left(z_{i}\right)\left[{\dot{e}}_{i}\left(t\right)+e_{i}\left(t\right)\right]\\ \; & =-2\beta \sum_{i=1}^{N}e_{i}^{T}\left(t\right)e_{i}\left(t\right)-\sum_{i=1}^{N}E_{i}^{T}\left(t\right)Q_{i}E_{i}\left(t\right)\\ \; & \;+2\beta \sum_{i=1}^{N}\left({\dot{e}}_{i}^{T}\left(t\right)+e_{i}^{T}\left(t\right)\right)\left[\eta_{i}-D_{i}^{-1}\left(z_{i}\right)\varepsilon_{i}\left(z_{i},{\dot{z}}_{i}\right)\right]\\ \; & \;-2\sum_{i=1}^{N}E_{i}^{T}\left(t\right)\left\{\beta \sigma D_{i}^{-1}\left(z_{i}\right)\Gamma H_{i}\left(z\right)-M_{i}\Theta_{i}\left(z_{i}\right)\right\}\left[{\dot{e}}_{i}\left(t\right)+e_{i}\left(t\right)\right]. \end{array}$$

Then, by substituting the control protocol (6) and the coupling matrix function (8) into Equation (14) and taking into consideration Assumption 1, the following expression is achieved:

(15) $$\begin{array}{cc} {\dot{V}}_{i}\left(t\right) & =-2\beta \sum_{i=1}^{N}e_{i}^{T}\left(t\right)e_{i}\left(t\right)-\sum_{i=1}^{N}E_{i}^{T}\left(t\right)Q_{i}E_{i}\left(t\right)\\ \; & \;+2\beta \sum_{i=1}^{N}\left({\dot{e}}_{i}^{T}\left(t\right)+e_{i}^{T}\left(t\right)\right)\left[\eta_{i}-D_{i}^{-1}\left(z_{i}\right)\varepsilon_{i}\left(z_{i},{\dot{z}}_{i}\right)\right]\\ \; & \leq -2\beta \sum_{i=1}^{N}e_{i}^{T}\left(t\right)e_{i}\left(t\right)-\sum_{i=1}^{N}E_{i}^{T}\left(t\right)Q_{i}E_{i}\left(t\right)\\ \; & \;+2\beta \sum_{i=1}^{N}∥{\dot{e}}_{i}^{T}\left(t\right)+e_{i}^{T}\left(t\right)∥∥D_{i}^{-1}\left(z_{i}\right)∥\omega \left(t\right)+2\beta \sum_{i=1}^{N}\left({\dot{e}}_{i}^{T}\left(t\right)+e_{i}^{T}\left(t\right)\right)\eta_{i}\\ \; & =-2\beta \sum_{i=1}^{N}e_{i}^{T}\left(t\right)e_{i}\left(t\right)-\sum_{i=1}^{N}E_{i}^{T}\left(t\right)Q_{i}E_{i}\left(t\right)\\ \; & \;+2\beta \sum_{i=1}^{N}∥{\dot{e}}_{i}^{T}\left(t\right)+e_{i}^{T}\left(t\right)∥∥D_{i}^{-1}\left(z_{i}\right)∥\omega \left(t\right)\\ \; & \;-2\beta \sum_{i=1}^{N}\left({\dot{e}}_{i}^{T}\left(t\right)+e_{i}^{T}\left(t\right)\right)∥D_{i}^{-1}\left(z_{i}\right)∥\omega \left(t\right)\tilde{sign}\left({\dot{e}}_{i}\left(t\right)+e_{i}\left(t\right)\right)\\ \; & =-2\beta \sum_{i=1}^{N}e_{i}^{T}\left(t\right)e_{i}\left(t\right)-\sum_{i=1}^{N}E_{i}^{T}\left(t\right)Q_{i}E_{i}\left(t\right)\leq 0. \end{array}$$

□

**Remark** **7.** *From inequality (15), it can be seen that $\dot{V}\left(t\right)$ is negative semi-definite about $e_{i}\left(t\right)$, ${\dot{e}}_{i}\left(t\right)$, and $E_{i}\left(t\right)$. In other words, the error systems $e_{i}\left(t\right)$, ${\dot{e}}_{i}\left(t\right)$, and $E_{i}\left(t\right)$ are stable; that is, $e_{i}\left(t\right)$, ${\dot{e}}_{i}\left(t\right)$, and $E_{i}\left(t\right)$ are bounded. By using Barbalat’s lemma [30,31,32], one can see thatthe position tracking error $e_{i}\left(t\right)=z_{i}\left(t\right)-z_{i}^{d}\left(t\right)$$\overset{t\to +\infty }{\to }$ 0 holds and the velocity vector ${\dot{z}}_{i}\in R{}^{n}$ and the outgoing communication strength vector $P_{i}=P_{i}\left(t\right)\in R{}^{N}$ are bounded for all $i\in \left\{1,2,\ldots ,N\right\}$*

**Remark** **8.** *The following procedures for applying Theorem 1 are summarized to achieve the position tracking control for the RMSs.*

Step 1. Give the desired joint position trajectory $z_{i}^{d}\left(t\right)\in R{}^{n}$, its derivatives ${\dot{z}}_{i}^{d}\left(t\right)\in R{}^{n}$, ${\ddot{z}}_{i}^{d}\left(t\right)\in R{}^{n}$ for RMS (3), the desired communication strength trajectory $P_{i}^{d}\left(t\right)\in R{}^{N}$ for CSS (4) for all $i\in \left\{1,2,\ldots ,N\right\}$, and their initial state values.

Step 2. Determine the known matrices $D_{i}\left(z_{i}\right)$, $C_{i}\left(z_{i},{\dot{z}}_{i}\right)$, the known vector $G_{i}\left(z_{i}\right)$, the common coupling strength σ, the diagonal matrix Γ, the inner coupling matrix $H_{i}\left(z\right)$, the known positive function ω(t), and the adjustable positive parameter β.

Step 3. Determine the positive definite matrices $M_{i}$ by solving the Lyapunov Equation (5).

Step 4. Determine the designed control protocols (6) and (7) and the coupling matrix function (8) by substituting in the above parameters. At this point, Theorem 1 is realized; that is, $e_{i}\left(t\right)=z_{i}\left(t\right)-z_{i}^{d}\left(t\right)$$\overset{t\to +\infty }{\to }$ 0 holds, and the outgoing communication strength vector $P_{i}=P_{i}\left(t\right)\in R{}^{N}$ is bounded for all $i\in \left\{1,2,\ldots ,N\right\}$.

## 4. Illustrative Example

**Figure 2:**
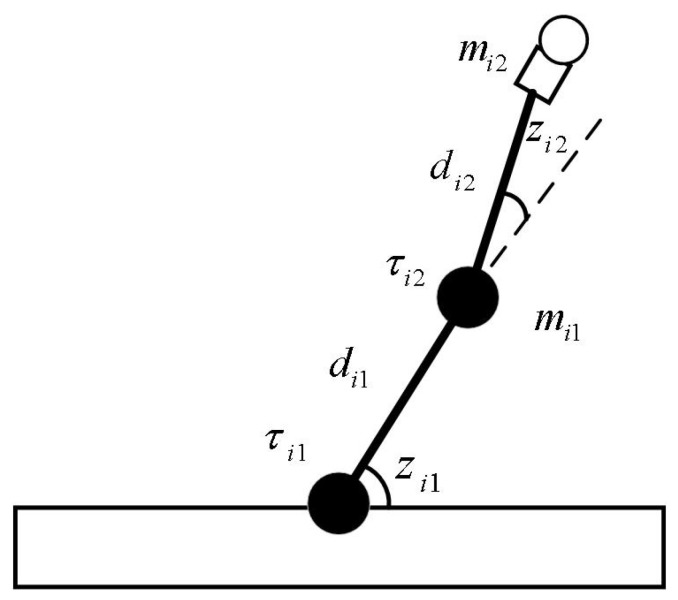
The mechanical model of *i*th two-link robotic manipulator.

In this section, we test our proposed control scheme on the position tracking control of *N* two-link (*n* = 2) robotic manipulators with uncertainties in [33] via simulation experiments, in which the dynamics model of each isolate robotic manipulator (see Figure 2) in joint space can be expressed as

(16) $$D_{i}\left(z_{i}\right){\ddot{z}}_{i}+C_{i}\left(z_{i},{\dot{z}}_{i}\right){\dot{z}}_{i}+G_{i}\left(z_{i}\right)+\varepsilon_{i}\left(z_{i},{\dot{z}}_{i}\right)=\tau_{i}$$

where $z_{i}={\left(z_{i1},z_{i2}\right)}^{T}\in R^{2}$ and ${\dot{z}}_{i}={\left({\dot{z}}_{i1},{\dot{z}}_{i2}\right)}^{T}\in R^{2}$ denote the position vector of the *i*th robot arm as well as the velocity vector, respectively, $z_{i1}$ denotes the angular position of the first robot arm, and $z_{i2}$ denotes the angular position of the second robot arm. In this paper, the main consideration is the position tracking problem of the two-link robotic manipulators, so the dynamics of $z_{i}$ is the main concern of this paper.

The internal connection relation term can be described as a given communication protocol. Inspired by the literature [21], we consider the given communication transmission protocol as $\sum_{j=1}^{N}p_{ij}\left(t\right)\Gamma h_{j}\left(z\right)$ in this paper. Then, Equation (16) can be rewritten in the form of Equation (1) in this paper, as follows:

(17) $$D_{i}\left(z_{i}\right){\ddot{z}}_{i}+C_{i}\left(z_{i},{\dot{z}}_{i}\right){\dot{z}}_{i}+G_{i}\left(z_{i}\right)+\varepsilon_{i}\left(z_{i},{\dot{z}}_{i}\right)=\tau_{i}+\sigma \sum_{j=1}^{N}p_{ij}\left(t\right)\Gamma h_{j}\left(z\right),$$

In addition, the dynamics of the communication strength $p_{ij}\left(t\right)$, $i,j\in \left\{1,2,\ldots ,N\right\}$, between the two-link robotic manipulators is shown below, which is the same as Equation (4).

(18) $${\dot{P}}_{i}\left(t\right)=A_{i}P_{i}\left(t\right)+\Theta_{i}\left(z_{i}\right)\left(z_{i}\left(t\right)+{\dot{z}}_{i}\left(t\right)\right)$$

In Figure 2, $m_{i1}$ and $m_{i2}$ denote the masses of the first arm and the second arm for the *i*th robotic manipulator, respectively. $d_{i1}$ and $d_{i2}$ denote the lengths of the first arm and the second arm for the *i*th robotic manipulator, respectively. $\tau_{i1}$ and $\tau_{i2}$ denote the torque on the first arm and the second arm for the *i*th robotic manipulator, respectively. $z_{i1}$ and $z_{i2}$ denote the positions of the first arm and the second arm for the *i*th robotic manipulator, where $i\in \left\{1,2,\ldots ,N\right\}$.

Note that $P\left(t\right)={\left[P_{1}^{T}\left(t\right),P_{2}^{T}\left(t\right),\ldots ,P_{N}^{T}\left(t\right)\right]}^{T}\in R{}^{N^{2}}$, $e\left(t\right)={\left[e_{1}^{T}\left(t\right),e_{2}^{T}\left(t\right),\ldots ,e_{N}^{T}\left(t\right)\right]}^{T}\in R{}^{nN}$, $E\left(t\right)={\left[E_{1}^{T}\left(t\right),E_{2}^{T}\left(t\right),\ldots ,E_{N}^{T}\left(t\right)\right]}^{T}\in R{}^{N^{2}}$, and $P^{d}\left(t\right)={\left[{\left(P_{1}^{d}\left(t\right)\right)}^{T},{\left(P_{2}^{d}\left(t\right)\right)}^{T},\ldots ,{\left(P_{1}^{d}\left(t\right)\right)}^{T}\right]}^{T}\in R{}^{N^{2}}$.

The numerical parameters of the *i*th two-link robotic manipulator are selected according to the following steps, which are derived from the numerical simulation example in [33].

(i) The inertia matrix $D_{i}\left(z_{i}\right)$, the Coriolis and centripetal force matrix $C_{i}\left(z_{i},{\dot{z}}_{i}\right)$, and the gravitational force vector $G_{i}\left(z_{i}\right)$ can be described as

$$D_{i}\left(z_{i}\right)=\left[\begin{array}{cc} m_{i1}d_{i1}^{2}+m_{i2}\left(d_{i1}^{2}+d_{i2}^{2}+2d_{i1}d_{i2}\cos z_{i2}\right) & m_{i2}d_{i2}^{2}+m_{i2}d_{i1}d_{i2}\cos z_{i2}\\ m_{i2}d_{i2}^{2}+m_{i2}d_{i1}d_{i2}\cos z_{i2} & m_{i2}d_{i2}^{2} \end{array}\right],$$

$$C_{i}\left(z_{i},{\dot{z}}_{i}\right)=\left[\begin{array}{cc} -2m_{i2}d_{i1}d_{i2}\sin z_{i2}{\dot{z}}_{i2} & -2m_{i1}d_{i1}d_{i2}\sin z_{i2}{\dot{z}}_{i2}\\ m_{i2}d_{i1}d_{i2}\sin z_{i2}{\dot{z}}_{i1} & 0 \end{array}\right],$$

$$G_{i}\left(z_{i}\right)=\left[\begin{array}{c} m_{i2}d_{i2}g\cos\left(z_{i1}+z_{i2}\right)+\left(m_{i1}+m_{i2}\right)d_{i1}g\cos\left(z_{i1}\right)\\ m_{i2}d_{i2}g\cos\left(z_{i1}+z_{i2}\right) \end{array}\right].$$

Moreover, the uncertain function $\varepsilon_{i}\left(z_{i},{\dot{z}}_{i}\right)$ can be described as

$$\varepsilon_{i}\left(z_{i},{\dot{z}}_{i}\right)=\left[\begin{array}{c} 0.5sign\left({\dot{e}}_{i1}\right)\left[0.1+\exp\left(-\left|{\dot{e}}_{i1}\right|\right)\right]\\ sign\left({\dot{e}}_{i2}\right)\left[0.1+\exp\left(-\left|{\dot{e}}_{i2}\right|\right)\right] \end{array}\right]+\left[\begin{array}{c} \gamma_{i}\\ \gamma_{i} \end{array}\right],$$

where $\gamma_{i}=randn\left(1\right)$ is a random number to reflect the uncertainty of the function $\varepsilon_{i}\left(z_{i},{\dot{z}}_{i}\right)$ and $\Gamma =diag\left(b_{1},b_{2}\right)\in R{}^{2\times 2}$ and $h_{j}=[\cos\left(x_{j1}\right),\sin\left(x_{j2}\right){]}^{T}\in R{}^{2}$, respectively, in which $b_{s}=randn\left(1\right)$, $i,j\in \left\{1,2,\ldots ,N\right\}$, $s\in \left\{1,2\right\}$ and randn(1) denotes the generation of a random number with a standard normal distribution.

(ii) The initial states of $z\left(0\right)\in R{}^{nN}$ and $P\left(0\right)\in R{}^{N^{2}}$ are generated by the functions rand(nN,1) rad and $rand(N^{2},1)$ rad in MATLAB, respectively. rand(a,b) denotes the generation of a matrix of size a×b, where each element is a random number in the interval [0, 1]. The desired joint position trajectory $z_{i}^{d}\left(t\right)\in R{}^{n}$ are $z_{i}^{d}\left(t\right)={\left[\sin\left(2\pi t\right),\cos\left(2\pi t\right)\right]}^{T}$, and the desired communication strength trajectory $P_{i}^{d}\left(t\right)\in R{}^{N}$ is given by Equation (9).

(iii) In order to generate the Hurwitz matrix $A_{i}$, let $\omega_{i}=-rand\left(1\right)\left(\omega_{i}\neq 0\right)$ be a randomly generated negative number and $W_{i}\in R{}^{N\times N}$ be a stochastically produced *N*-order invertible symmetric matrix in MATLAB. It can be concluded that the Hurwitz matrix $A_{i}=W_{i}diag\left\{w_{1},w_{2},\ldots ,w_{N}\right\}W_{i}^{-1}$.

(iv) Let $Q_{i}=\phi I_{N}\in R{}^{N\times N}$, in which ϕ=5rand(1) and $I_{N}$ denotes the *N* order identity matrix, which is substituted into the Lyapunov equation, Equation (5), to obtain the positive definite matrix $M_{i}\in R{}^{N\times N}$, $i\in \left\{1,2,\ldots ,N\right\}$.

**Figure 3:**
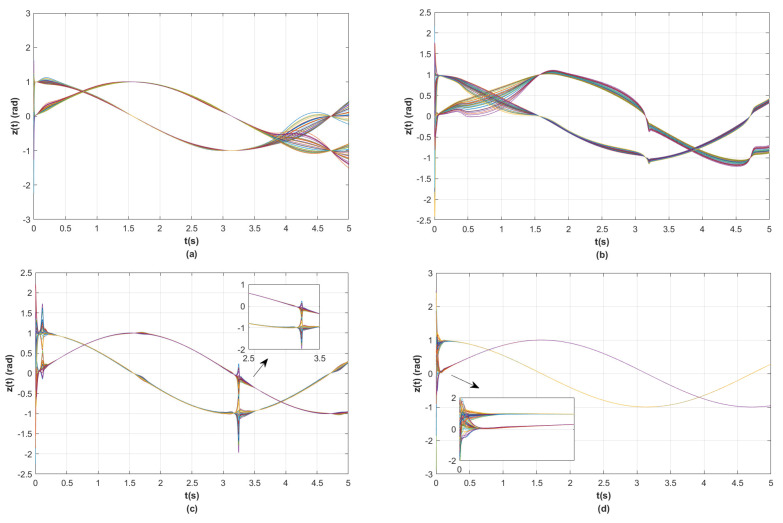
(**a**) The state curves of position for RMS under the control scheme from [33]; (**b**) the state curves of position for RMS under the control scheme from [34]; (**c**) the state curves of position for RMS under the control scheme from [35]; (**d**) the state curves of position for RMS under the control scheme from this paper.

**Figure 4:**
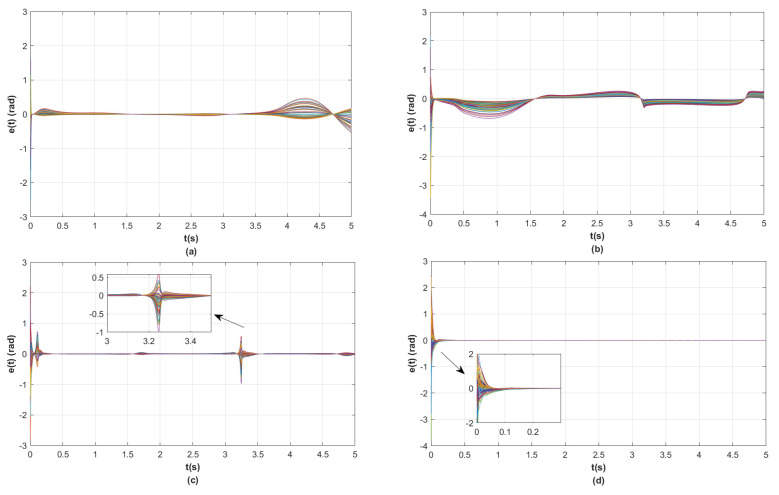
(**a**) The position error curves of RMS under the control scheme from [33]; (**b**) the position error curves of RMS under the control scheme from [34]; (**c**) the position error curves of RMS under the control scheme from [35]; (**d**) the position error curves of RMS under the control scheme from this paper.

**Figure 5:**
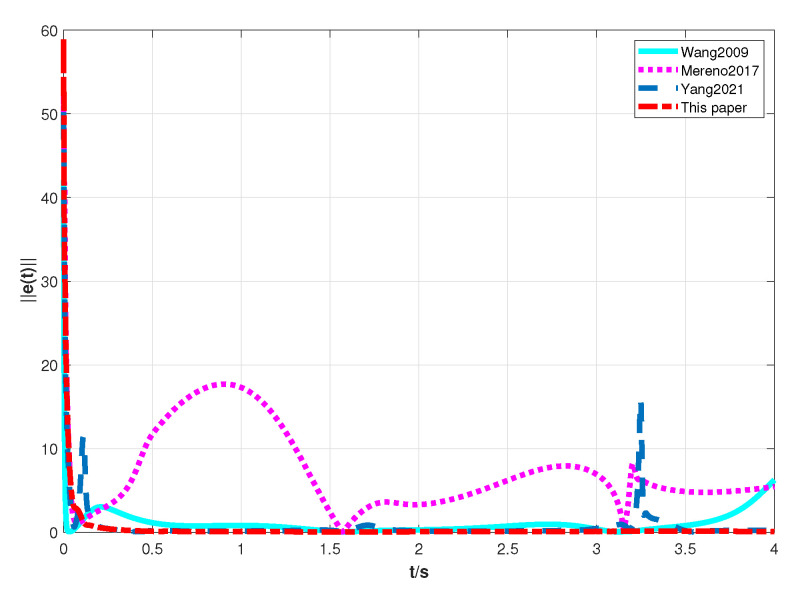
The response curves of $∥e(t)∥$ with the control scheme in [33,34,35] and this paper.

**Figure 6:**
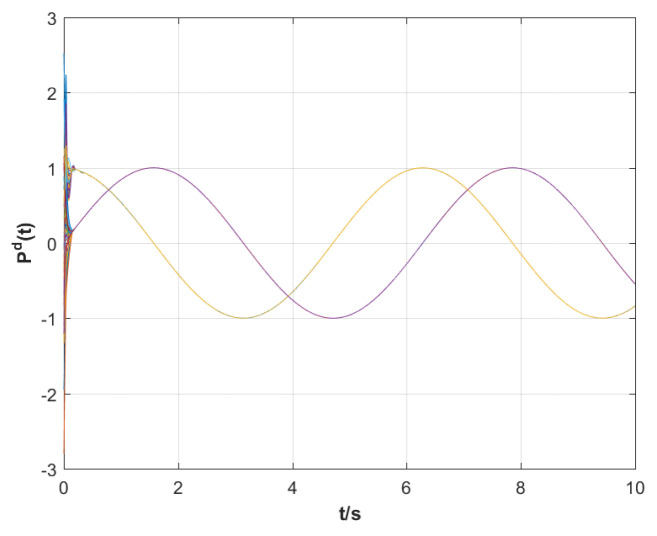
The response curves of the desired communication strength trajectory $P^{d}$ for CSS.

In the simulations, the model parameters of the two-link robotic manipulator are N=30, $m_{i1}$ = 10 kg, $m_{i2}$ = 2 kg, $d_{i1}=1.1$ m, $d_{i2}=0.8$ m, *g* = 9.8 m/s^2^, σ=5.7, and β=5randn(1). Finally, the model parameters and the matrices obtained from the above steps are substituted into the position tracking control protocols $\tau_{i}$ (6) and $\eta_{i}$ (7) and the coupling matrix function $\Theta_{i}\left(z_{i}\right)$ (8) designed in this paper. In addition, in order to demonstrate the advantages of the position tracking control scheme synthesized in this paper, we introduce an experiment comparing the position tracking proposed in [33,34,35] with ours. For simplicity, let $∥e(t)∥=\sqrt{\sum_{i=1}^{N}{∥e_{i}\left(t\right)∥}^{2}}$ be the total position tracking error of the RMS in the MRMS. The simulation results are shown in Figure 3, Figure 4, Figure 5 and Figure 6.

The following analytical results can be derived from the simulation results in Figure 3, Figure 4, Figure 5 and Figure 6.

(i) As shown in Figure 3, the differently colored curves show the state of the joint angular position of the 30 robotic manipulators. It can be seen that the control scheme in [33,34,35] cannot make the joint angle positions of the RMS track the desired trajectory. That is to say, the control scheme in [33,34,35] leads to larger errors of position tracking for the RMS, but the control scheme in this paper can make the position state vector of the MRMS track the desired joint position trajectory quickly.

(ii) Figure 4 and Figure 5 illustrate that the results of the position tracking control scheme for the RMS in this paper are comparative with the ones in [33,34,35]. It is clear from Figure 4 that the position tracking control error of the RMS converges asymptotically to zero by using the control scheme in this paper. Nevertheless, the position tracking control error of the RMS does not tend to zero when employing the control scheme in [33,34,35]. Moreover, the fast convergence speed of the position tracking control error of RMS in this paper is faster than the ones in [33,34,35]. Above all, it can be seen that when realizing the position tracking control of the RMS, the control scheme of this paper is more appropriate than the ones in [33,34,35].

(iii) It is clear from Figure 6 that the desired communication strength trajectory $P^{d}$ is bounded and does not converge asymptotically to zero, which means that the eventual MRMS structure is shown as all the RMSs are not isolated when the position tracking of the RMS happens.

## 5. Conclusions

Position tracking control of the RMS has been achieved for a class of uncertain MRMSs with communication strength dynamics by employing the designed tracking control protocol of the RMS and the coupling matrix function of the CSS in this paper. Compared to the existing results about the position tracking of the RMS, the advantages of this paper are that the MRMS with uncertainties can be considered as the composition system with the RMS and the CSS, and both subsystems are coupled to each other. Moreover, the dynamical model of the RMS is shown mathematically as a vector differential equation with the second derivative term for the more suitable applications in engineering practice. From the number simulation results, it can be seen that the control scheme designed in this paper can effectively control the state of the RMS and CSS to track the given reference trajectories. In the future, the velocity tracking problem and the double-tracking of position and velocity for MRMS will be considered by designing a new control scheme under communication topology constrained bit rates [36,37] or attacks [38,39].

## Data Availability

Data are contained within the article.

## Abbreviations

The following abbreviations are used in this manuscript:

MRMS	multiple robotic manipulator system
RMS	robotic manipulator subsystem
CSS	communication strength subsystem

## References

[1] Gueaieb W., Al-Sharhan S., Bolic M. (2007). Robust computationally efficient control of cooperative closed-chain manipulators with uncertain dynamics. Automatica.

[2] Gueaieb W., Karray F., Al-Sharhan S. (2007). A robust hybrid intelligent position/force control scheme for cooperative manipulators. IEEE-ASME Trans. Mech..

[3] Nijmeijer H., Rodriguez-Angeles A. (2003). Synchronization of Mechanical Systems.

[4] Zhang Y.H., Jiang Y.L., Zhang W.L., Ai X.L. (2022). Distributed coordinated tracking control for multi-manipulator systems under intermittent communications. Nonlinear Dyn..

[5] Gudiño-Lau J., Arteaga M.A. (2005). Dynamic model and simulation of cooperative robots: A case study. Robotica.

[6] Martínez-Rosas J.C., Arteaga M.A., Castillo-Sánchez A.M. (2006). Decentralized control of cooperative robots without velocity–force measurements. Automatica.

[7] Hamedani M.H., Zekri M., Sheikholeslam F., Selvaggio M., Ficuciello F., Siciliano B. (2021). Recurrent fuzzy wavelet neural network variable impedance control of robotic manipulators with fuzzy gain dynamic surface in an unknown varied environment. Fuzzy Sets Syst..

[8] Purwar S., Kar I.N., Jha A.N. (2005). Adaptive control of robot manipulators using fuzzy logic systems under actuator constraints. Fuzzy Sets Syst..

[9] Liu M., Zhang J.Z., Shang M.S. (2022). Real-time cooperative kinematic control for multiple robots in distributed scenarios with dynamic neural networks. Neurocomputing.

[10] Zhao D.Y., Li S.Y., Zhu Q.M. (2016). Adaptive synchronised tracking control for multiple robotic manipulators with uncertain kinematics and dynamics. Int. J. Syst. Sci..

[11] Gao P.T., Wang Y.H., Peng Y., Zhang L.L., Li S.P. (2023). Tracking control of the nodes for the complex dynamical network with the auxiliary links dynamics. Inform. Sci..

[12] Sun D. (2003). Position synchronization of multiple motion axes with adaptive coupling control. Automatica.

[13] Abdessameud A., Polushin I.G., Tayebi A. (2013). Synchronization of Lagrangian systems with irregular communication delays. IEEE Trans. Autom. Control.

[14] Zhao D.Y., Ni W., Zhu Q.M. (2014). A framework of neural networks based consensus control for multiple robotic manipulators. Neurocomputing.

[15] Nuño E., Aldana C.I., Basañez L. (2017). Task space consensus in networks of heterogeneous and uncertain robotic systems with variable time-delays. Int. J. Adapt. Control.

[16] Alinezhad H.S., Esfanjani R.M. (2022). Nonlinear H∞ control for synchronization of networked manipulators subject to delayed communication. J. Franklin Ins..

[17] Fan Y.Q., An Y., Wang W.Q., Yang C.G. (2020). TS fuzzy adaptive control based on small gain approach for an uncertain robot manipulators. Int. J. Fuzzy Syst..

[18] Fan Y.Q., Kang T.T., Wang W.Q., Yang C.G. (2019). Neural adaptive global stability control for robot manipulators with time-varying output constraints. Int. J. Robust Nonlin..

[19] Kumar P., Zaheer A. (2019). Ego-network stability and innovation in alliances. Acad. Manag. J..

[20] Liu C.L., Wang J.Q., Zhang H. (2018). Spatial heterogeneity of ports in the global maritime network detected by weighted ego network analysis. Marit. Policy Manag..

[21] Gao P.T., Wang Y.H., Liu L.Z., Zhang L.L., Tang X. (2021). Asymptotical state synchronization for the controlled directed complex dynamic network via links dynamics. Neurocomputing.

[22] Wang Y.H., Wang W.L., Zhang L.L. (2020). State synchronization of controlled nodes via the dynamics of links for complex dynamical networks. Neurocomputing.

[23] Zhao J.X., Wang Y.H., Gao P.T., Zhang L.L., Wang X.X. (2023). Bipartite Tracking Control for the Complex Dynamical Networks Based on Links Dynamics. 2023 9th International Conference on Control Science and Systems Engineering (ICCSSE).

[24] Davison E.J. (1977). Connectability and structural controllability of composite systems. Automatica.

[25] Wang Y.H., Zhang S.Y. (2000). Robust control for nonlinear similar composite systems with uncertain parameters. IEEE Proc.-Control Theory Appl..

[26] Chen C.T., Desoer C. (1967). Controlability and observability of composite systems. IEEE T. Autom. Control.

[27] Zhao D.Y., Zhu Q.M. (2014). Position synchronised control of multiple robotic manipulators based on integral sliding mode. Int. J. Syst. Sci..

[28] Cui R.X., Yan W.S. (2012). Mutual synchronization of multiple robot manipulators with unknown dynamics. J. Intell. Robot. Syst..

[29] Zhang J.Z., Jin L., Yang C.G. (2021). Distributed cooperative kinematic control of multiple robotic manipulators with an improved communication efficiency. IEEE-ASME Trans. Mech..

[30] Gao P.T., Wang Y.H., Peng Y., Zhang L.L., Huang Y.Y. (2022). Tracking control for the nonlinear complex dynamical network assisted with outgoing links dynamics. Int. J. Robust Nonlin..

[31] Wang Y.H., Fan Y.Q., Wang Q.Y., Zhang Y. (2012). Stabilization and synchronization of complex dynamical networks with different dynamics of nodes via decentralized controllers. IEEE Trans. Circuits Syst. I.

[32] Gao P.T., Wang Y.H., Zhao J.X., Zhang L.L., Peng Y. (2023). Links synchronization control for the complex dynamical network. Neurocomputing.

[33] Wang Y.N., Sun W., Xiang Y.Q., Miao S.Y. (2009). Neural network-based robust tracking control for robots. Intell. Autom. Soft Comput..

[34] Moreno-Valenzuela J., Aguilar-Avelar C., Puga-Guzmán S., Santibáñez V. (2017). Two adaptive control strategies for trajectory tracking of the inertia wheel pendulum: Neural networks vis à vis model regressor. Intell. Autom. Soft Comput..

[35] Yang Y.L., Ding Z.H., Wang R., Modares H., Wunsch D.C. (2021). Data-driven human-robot interaction without velocity measurement using off-policy reinforcement learning. IEEE-CAA J. Autom..

[36] Li J.Y., Wang Z.D., Lu R.Q., Xu Y. (2022). Distributed filtering under constrained bit rate over wireless sensor networks: Dealing with bit rate allocation protocol. IEEE T. Autom. Control.

[37] Song J., Wang Y.K., Niu Y.G. (2022). Dynamic event-triggered terminal sliding mode control under binary encoding: Analysis and experimental validation. IEEE Trans. Circuits Syst. I.

[38] Lu A.Y., Yang G.H. (2019). Secure switched observers for cyber-physical systems under sparse sensor attacks: A set cover approach. IEEE Trans. Autom. Control.

[39] Wang J.L., Wang L., Wu H.N. (2022). Synchronization for complex networks with multiple state or delayed state couplings under recoverable attacks. IEEE Trans. Syst. Man Cybern..

